# Supplementary material for: Metabolomic Analysis of Murine Tissues Infected with Brucella melitensis
Source: bioRxiv. 2024 Nov 16:2024.11.16.623915. Preprint. [Version 1] doi: 10.1101/2024.11.16.623915 (PMC11601316; doi:10.1101/2024.11.16.623915)
Supplement: Supplement 1 [file NIHPP2024.11.16.623915v1-supplement-1.pdf]

bioRxiv preprint doi: https://doi.org/10.1101/2024.11.16.623915; this version posted November 16, 2024. The copyright holder for this preprint (which was not certified by peer review) is the author/funder, who has granted bioRxiv a license to display the preprint in perpetuity. It is made available under aCC-BY 4.0 International license.

Supporting Information Captions

Table S1: Primers used in this study.

bioRxiv preprint doi: https://doi.org/10.1101/2024.11.16.623915; this version posted November 16, 2024. The copyright holder for this preprint (which was not certified by peer review) is the author/funder, who has granted bioRxiv a license to display the preprint in perpetuity. It is made available under aCC-BY 4.0 International license.

Table S2.1: Metabolite levels in spleens from naïve mice, and from spleens at 7, 14, and 28 days post-infection with *B. melitensis*.

Table S2.2: Metabolite levels in livers from naïve mice, and from livers at 7, 14, and 28 days post-infection with *B. melitensis*.

Table S2.3: Metabolite levels in reproductive tracts from naïve female mice, and from reproductive tracts at 7, 14, and 28 days post-infection with *B. melitensis*.

Table S3.1: Metabolite levels in spleens from naïve mice, and from spleens at 7 days post-infection with *B. melitensis*.

Table S3.2: Metabolite levels in livers from naïve mice, and from livers at 7 28 days post-infection with *B. melitensis*.

Table S3.3: Metabolite levels in reproductive tracts from naïve female mice, and from reproductive tracts at 7 days post-infection with *B. melitensis*.
